# Nano‐optogenetic immunotherapy

**DOI:** 10.1002/ctm2.1020

**Published:** 2022-09-13

**Authors:** Kai Huang, Xiaoxuan Liu, Gang Han, Yubin Zhou

**Affiliations:** ^1^ Department of Biochemistry and Molecular Biotechnology University of Massachusetts Chan Medical School Worcester Massachusetts USA; ^2^ Center for Translational Cancer Research Institute of Biosciences and Technology Texas A&M University Houston Texas USA; ^3^ Department of Translational Medical Sciences School of Medicine Texas A&M University Houston Texas USA

**Keywords:** biophotonics, cancer, CAR T cell, immunotherapy, nanotechnology, optogenetics

## Abstract

Chimeric antigen receptor (CAR) T cell‐based immunotherapy has been increasingly used in the clinic for cancer intervention over the past 5 years. CAR T‐cell therapy takes advantage of genetically‐modified T cells to express synthetic CAR molecules on the cell surface. To date, up to six CAR T cell therapy products have been approved by the Food and Drug Administration for the treatment of leukaemia, lymphoma, and multiple myeloma. In addition, hundreds of CAR‐T products are currently under clinical trials to treat solid tumours. In both the fundamental research and clinical applications, CAR T cell immunotherapy has achieved exciting progress with remarkable remission or suppression of cancers. However, CAR T cell‐based immunotherapy still faces significant safety issues, as exemplified by “on‐target off‐tumour” cytotoxicity due to lack of strict antigen specificity. In addition, uncontrolled massive activation of infused CAR T cells may create severe systemic inflammation with cytokine release syndrome and neurotoxicity. These challenges call for a need to combine nanotechnology and optogenetics with immunoengineering to develop spatiotemporally‐controllable CAR T cells, which enable wireless photo‐tunable activation of therapeutic immune cells to deliver personalised therapy in the tumour microenvironment.

## 1 EXTERNAL STIMULI‐RESPONSIVE CELLULAR IMMUNOTHERAPY

Despite the exciting progess of CAR T cell immunotherapy in both fundamental research and clinical applications,^1^, ^2^ it is still suffering from safety concerns associated with uncontrollable CAR T cell activities. Recent advances in nanotechnology and immunoengineering provide versatile approaches for the remote control of CAR T cell activities. For example, by incorporating genetically‐encoded light‐ or heat‐sensitive modules into immune cells, researchers are able to produce CAR T cells that can perceive external stimuli, such as light and ultrasound.^3^, ^4^, ^5^, ^6^, ^7^, ^8^, ^9^ The control of CAR T cells has been achieved either by (1) stimuli‐initiated transcription of the CAR transgene, or (2) stimuli‐driven reassembly of a split‐CAR on cell surface. In the first strategy, the transcription of the CAR gene will only be initiated by a specific stimulus, thus making the CAR expression remotely controllable. For instance, Pan et al. developed a genetic device to initiate the CAR expression by ultrasound.^5^ They applied a mechanical‐sensitive cation channel (Piezo1) to perceive the ultrasound vibration, which then initiates the Ca^2+^/NFAT‐responsive elements to drive CAR expression. Huang et al. developed a light‐inducible nuclear translocation and dimerization (LINTAD) system to control the CAR expression by blue‐light‐initiated nuclear translocation of transcriptional activators for inducible gene transcription.^6^ In two additional studies, Miller et al. and Wu et al. took advantage of heat shock‐responsive promoter to initiate CAR gene transcription under photothermal or ultrasound‐induced heat, respectively.^7^, ^8^ Although considerable suppression of the toxicity was demonstrated in these studies, the control over CAR T cell activity by the CAR expression is sub‐optimal because of the unsatisfactory spatiotemporal precision. Also, the CAR expression takes a long time, ranging from several hours to even days, thus remaining largely irreversible and sacrificing the real‐time control of CAR T cells. With regard to thermal control, heat shock‐responsive promoter might be easily perturbed by various environmental factors, including hypoxia or mechanical stress, making the controllability less specific. In contrast, stimuli‐driven reassembly of the split‐CAR provides reversible control of CAR T cell activities with superior spatial and temporal precision. In this strategy, the signalling domains of CAR are split into two parts and stay non‐functional at the resting condition. On each split part, a stimulation‐responsive dimerizer was installed, which could drive the reassembly of the split‐CAR under a specific stimulus, thus making the re‐assembled CAR fully functional with tumour‐killing ability.^9^

## 2 NANO‐OPTOGENETICS FOR WIRELESS CONTROL OF IMMUNOTHERAPY

**FIGURE 1:**
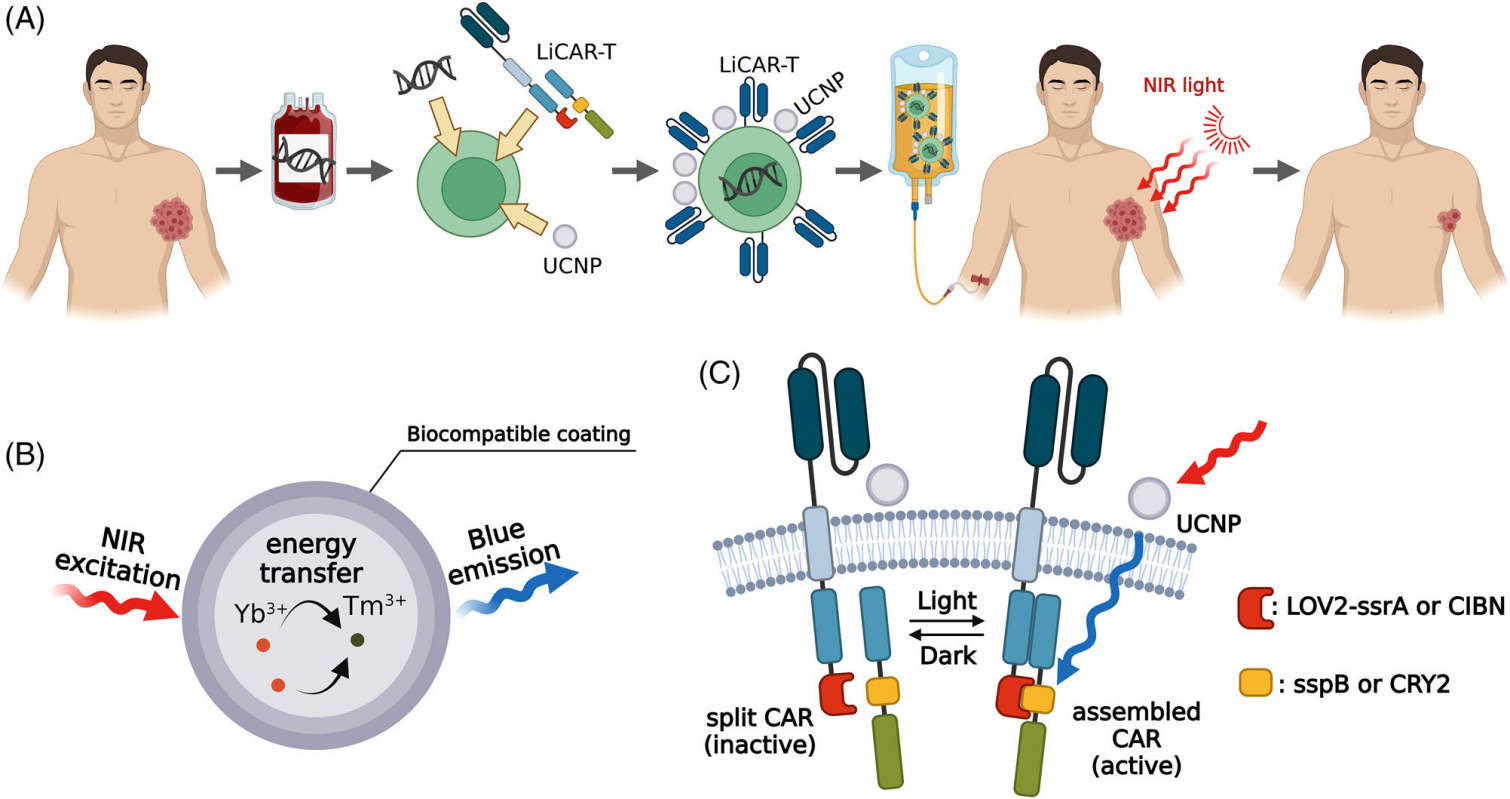
Schematic of UCNP‐mediated nano‐optogenetic cellular immunotherapy by using light‐switchable CAR T (LiCAR‐T) cells. (A) The general practice of using LiCAR‐T‐UCNP for wirelessly controlled nano‐optogenetic immunotherapy. (B) Schematic of UCNP as the nanotransducer to convert NIR light into blue light. (C) Schematic of UCNP‐mediated wireless control of LiCAR‐T cells by NIR stimulation. The signalling domains of CAR are split into two separate polypeptides, with each appended with one component from a pair of optical dimerizers (either LOV2‐ssrA/sspB or CRY2/CIBN). UCNPs sever as the nanotransducer to convert NIR excitation into blue emission via energy transfer and photon‐upconversion from Yb^3+^ to Tm^3+^. The blue emission initiates the assembly of optical dimerizer and brings the two CAR splits into close proximity, thus enabling a functional CAR reassembly to restore efficient tumour‐killing ability

Based on the light‐stimulated reassembly of the split‐CAR design, Nguyen and Huang et al. developed a light‐switchable CAR T cell (LiCAR‐T) system by using a light‐oxygen‐voltage (LOV) domain‐based optical dimerizer (LOV2‐ssrA/sspB) or cryptochrome 2 with CRY‐interacting basic helix‐loop‐helix N (CRY2/CIBN) as the light‐responsive dimerization tools for the optogenetic control of CAR T cell immunotherapy (Figure 1).^10^ The authors optimised the design of the LiCAR‐T system to solve several technical issues, including poor plasma membrane targeting, undesired nuclear accumulation, and background activation in dark condition. To validate the spatiotemporal control of the tumour‐killing activity of LiCAR‐T cells, the authors performed a series of in vitro co‐culturing experiments with CD19‐positive tumour cells. To solve the limited tissue penetration issue associated with blue light‐responsive tools, Nguyen et al. further applied upconversion nanoparticles or plates (UCNPs) as the nanotransducer to perceive deep tissue‐penetrating near‐infrared (NIR, 700–1000 nm) light as the wireless stimulation. UCNPs are a type of unique luminescent nanomaterials that convert NIR excitation into visible light with shorter wavelengths (Figure 1). Taking advantage of abundant metastable intermediate energy levels in lanthanide ions, UCNPs provide ladder‐like energy diagrams for electrons to jump up multiple steps by perceiving multiple excitation photons. The authors further engineered the dopant and size of UCNPs to obtain 4.5 times enhanced NIR‐to‐blue upconverted light emission, which allows researchers to use the NIR light as the wireless stimulus to excite the injected UCNPs and obtain efficient blue emission to drive the reassembly of split‐CAR.^10^ Considering the fine spatial and temporal precision of both the NIR irradiation and the reversible split‐CAR design, the authors were able to restrict the LiCAR‐T cells activation strictly at the tumour sites under real‐time NIR stimulation. With this clever design, researchers can achieve effective tumour eradication without causing severe safety issues in tumour‐bearing mice, as indicated by substantial attenuation of B cell aplasia and reduced production of interleukin 6, a proinflammatory cytokine regarded as the culprit for CRS. Moreover, since the size and surface modification of UCNPs are highly tunable, the group also demonstrated intravenous administration of the LiCAR‐T‐UCNP conjugates for spatiotemporal control of anti‐tumour immunity in mouse models of tumour, clearing obstacles for future clinical translations.^10^

## 3 FUTURE PERSPECTIVES

Nano‐optogenetic immunotherapy promises to overcome the significant challenge of safety issues obstructing conventional CAR T cell immunotherapy by enabling spatiotemporal control of CAR T cell activities. Looking forward, we envision that nano‐optogenetic technologies can provide versatile approaches for wireless and spatiotemporal control of cellular immunotherapy with deeper tissue penetration. For instance, by engineering the UCNPs with efficient excitation at the second window NIR (1000–1700 nm), UCNP‐mediated optogenetics can reach even deeper tissues because of less scattering and less absorption in this photoactivation window. Moreover, the emerging mechanoluminescence nanomaterials, which can convert ultrasound stimulation into light output, are also promising to serve as the nanotransducer for ultrasound‐induced reassembly of split‐CAR with spatiotemporal and reversible control of therapeutic immune cells. In a similar manner, X‐ray‐excitable scintillation nanomaterials, which convert X‐ray stimulation into light, are also desirable to enable X‐ray‐controllable cellular immunotherapy with unlimited depth of tissue penetration.

The potential for nano‐optogenetic immunotherapy seems to be quite promising, but one has to keep in mind several caveats during clinical translations. First, it is always desirable to develop external stimuli and nanotransducers with better biocompatibility and efficiency, as well as deeper tissue penetration. For instance, light‐responsive CAR T cells are most sensitive to blue light, which requires the use of inorganic UCNPs to convert NIR into blue light for wireless activation in deeply‐buried tissues. Although studies have demonstrated that UCNPs with appropriate surface modifications do not cause acute toxicities during the therapeutic window, there remains a need for long‐term assessment of the biosafety of the nanomaterials. In this regard, biodegradable organic nanomaterials, such as the triplet‐triplet annihilation UCNPs (TTA‐UCNPs), might serve as an attractive. Second, nano‐optogenetic immunotherapy awaits further optimizations for the efficient treatment of solid tumours. Currently, FDA‐approved CAR T cell therapy products are only targeting haematological neoplasms. However, the potency of CAR T cell therapy for the solid tumour is less efficient due to several obstacles, such as the physical barrier (e.g., stromal cell and extracellular matrix) hindering the infiltration and trafficking of CAR T cells, the hypoxia and immunosuppressive tumour microenvironment (TME), as well as the heterogeneous antigen expression lowering the antigen‐recognition by CAR T cells. In this regard, nano‐optogenetic approaches can be designed with the ability to soften the physical barriers, alter the TME, and upregulate antigen presenting in solid tumours. Third, developing cost‐effective nano‐optogenetic immunotherapy is important for clinical applications. The commercially available CAR T products are autologous CAR T cells, which are manufactured based on the T cells collected from each individual patient. This personalised production of CAR T cells imposes a great financial burden on the patient and society, which generally costs more than 350 000 dollars for a single treatment. In contrast, allogeneic CAR T cells allow massive manufacturing to substantially reduce the overall cost of cellular immunotherapy. Moreover, it can provide “off‐the‐shelf” products that are immediately ready for use, in stark contrast to autologous CAR T cells requiring more than 10 days for ex vivo culture and expansion. Also, it is of great merit to address the limitations of the “graft‐versus‐host” safety issue of allogeneic CAR T cells by restricting the nano‐optogenetic immunotherapy localised at the tumour sites. Regardless of these potential problems, nano‐optogenetic immunotherapy provides spatiotemporal, wireless, and reversible control of CAR T cells for efficient and safer immunotherapy, thus holding great potential for the future development of next‐generation precision cancer treatment, in which the ‘living drug’ will be personalised on‐demand to each patient's particular situation.

## CONFLICT OF INTEREST

The authors declare no conflict of interest.
